# Report of the first AKI Round Table meeting: an initiative of the ESICM AKI Section

**DOI:** 10.1186/s40635-019-0280-z

**Published:** 2019-12-07

**Authors:** M. Ostermann, A. Schneider, T. Rimmele, I. Bobek, M. van Dam, M. Darmon, L. Forni, O. Joannes-Boyau, M. Joannidis, M. Legrand, J. Prowle, A. Zarbock, E. Hoste

**Affiliations:** 1 Department of Critical Care, King’s College London, Guy’s & St Thomas’ Hospital, London, UK; 2 Adult Intensive Care Unit, Centre Hospitalier Universitaire Vaudois (CHUV), Lausanne, Switzerland; 3 Department of Anesthesiology and Critical Care Medicine, Edouard Herriot Hospital, Hospices Civils de Lyon, Lyon, France; 4 Aneszteziológiai és Intenzív Terápiás Klinika, Semmelweis Egyetem, Budapest, Hungary; 5 Department of Intensive Care Medicine, University Medical Center Utrecht, Utrecht, The Netherlands; 6 Medical ICU, Saint-Louis University Hospital, AP-HP, Paris, France; 7 Department of Clinical and Experimental Medicine, Faculty of Health Sciences, University of Surrey and Intensive Care Unit, Royal Surrey County Hospital NHS Foundation Trust, Guildford, UK; 8 Service d’Anesthesie-Reanimation SUD, CHU de Bordeaux, Hôpital Magellan, Bordeaux, France; 9 Division of Intensive Care and Emergency Medicine, Department of Internal Medicine, Medical University Innsbruck, Innsbruck, Austria; 10 Department of Anesthesiology and Peri-operative Care, University of California, San Francisco, USA; 11 Critical Care and Perioperative Medicine Research Group, Adult Critical Care Unit, The Royal London Hospital, Barts Health NHS Trust, London, and William Harvey Research Institute, Queen Mary University of London, London, UK; 12 Department of Anesthesiology, Intensive Care and Pain Medicine, University Hospital Münster, Münster, Germany; 13 Intensive Care Unit, Ghent University Hospital, Ghent University, Ghent, Belgium; 14 Research Foundation-Flanders (FWO), Brussels, Belgium

## Abstract

**Purpose:**

Critical Care Nephrology is an emerging sub-specialty of Critical Care. Despite increasing awareness about the serious impact of acute kidney injury (AKI) and renal replacement therapy (RRT), important knowledge gaps persist. This report represents a summary of a 1-day meeting of the AKI section of the European Society of Intensive Care Medicine (ESICM) identifying priorities for future AKI research.

**Methods:**

International Members of the AKI section of the ESICM were selected and allocated to one of three subgroups: “AKI diagnosis and evaluation”, “Medical management of AKI” and “Renal Replacement Therapy for AKI.” Using a modified Delphi methodology, each group identified knowledge gaps and developed potential proposals for future collaborative research.

**Results:**

The following key research projects were developed: Systematic reviews: (a) epidemiology of AKI with stratification by patient cohorts and diagnostic criteria; (b) role of higher blood pressure targets in patients with hypertension admitted to the Intensive Care Unit, and (c) specific clearance characteristics of different modalities of continuous renal replacement therapy (CRRT).

Observational studies: (a) epidemiology of critically ill patients according to AKI duration, and (b) current clinical practice of CRRT.

Intervention studies:( a) Comparison of different blood pressure targets in critically ill patients with hypertension, and (b) comparison of clearance of solutes with various molecular weights between different CRRT modalities.

**Conclusion:**

Consensus was reached on a future research agenda for the AKI section of the ESICM.

## Introduction

Acute kidney injury (AKI) is common during critical illness and associated with serious short and long-term complications as well as increased use of health care resources [1–5]. Furthermore, managing AKI is a challenge worldwide and one that requires multidisciplinary input [6]. In the last decade, tremendous research efforts have been made and we have vastly improved our understanding of the complexity of AKI but many important questions regarding AKI remain unresolved [7, 8]. For instance, despite the increasing number of epidemiological studies highlighting the incidence and outcomes of AKI, this has not translated into any robust strategies to improve patient-centred outcomes. Undoubtedly, the heterogenous nature of AKI has contributed to the challenges of developing effective therapies.

The AKI section of the European Society of Intensive Care Medicine (ESICM) consists of health care professionals from different countries with a mutual interest in adult critical care nephrology. One of their key objectives is to support research and to facilitate clinical and academic collaborations. This approach has already led to successful funding applications, publications in high-impact journals and ongoing research projects, for instance, AKI-Epi [1], PEACE study (ClinicalTrials.gov Identifier: NCT02341885), REVERSE-AKI (ClinicalTrials.gov Identifier: NCT03251131), and PREV-AKI 2 (ClinicalTrials.gov Identifier: NCT03244514).

Clinical practice remains heterogenous and quality standards are lacking. In recognition of ongoing uncertainties and variation in clinical practice, the ESICM AKI section convened an AKI Round Table (ART) meeting. The objectives of this 1-day meeting were to determine priorities for future AKI research to be conducted under the umbrella of the AKI section. This report represents a summary of the conclusions (Table 1).

**Table 1:** Summary of research projects proposed by the ART panel

	Epidemiology of AKI	Medical therapy of AKI	Continuous renal replacement therapy
Main research question	What is the epidemiology of critically ill patients with AKI of different durations?	Is a higher target MAP in critically ill patients with pre-existing hypertension renoprotective?	What is the difference in large molecule clearance between convection versus diffusion?
Systematic review of literature	AKI prevalence	Role of higher MAP for patients with hypertension at risk of development or progression of AKI	Solute clearance in CVVH vs CVVHD
Cohort study	Retrospective cohort	–	International survey
RCT	–		
Target group		Hypertensive adult patients admitted to ICU	Patients on CRRT
Intervention group		Target MAP 80–90 mmHg for at least 48 hours after randomization	treatment with CVVH
Comparator		Target MAP 65-75 mmHg for at least 48 hours after randomization	Treatment with CVVHD
Primary outcome		In patients without AKI at randomization: Primary outcome: prevention of AKI in 7 days In patients with AKI and no need for RRT at randomization: Primary outcome: MAKE at 28 days	Clearance of beta 2 microglobulin
Sample size calculation		*Hypothesis 1:* Expecting 40% of MAKE 30 in patients with AKI in the control group and a 10% absolute decrease in the risk (ie. 30% of MAKE 30 in intervention group) Target: 182 patients per group (alpha risk 5%, power 80%) Target: 240 patients per group (alpha risk 5%, power 90%) *Hypothesis 2:* Expecting 40% of MAKE 30 in patients with AKI in the control group and a 5% absolute decrease in the risk (ie 35% of MAKE 30 in intervention group) Target: 742 patients per group (alpha risk 5%, power 80%) Target: 988 patients per group (alpha risk 5%, power 90%)	60 patients per group

*AKI* acute kidney injury, *ART* acute kidney injury round table, *CRRT* continuous renal replacement therapy, *CVVHD* continuous veno-venous haemodialysis, *CVVH* continuous veno-venous haemofiltration, *MAKE 30* major adverse kidney events at day 30, *MAP* mean arterial pressure, *RCT* randomized controlled trial

## Methods

### ART panel

The Chairs of the AKI section selected current members to join the first ART Meeting. Nomination was based on expertise and academic track record in the field of AKI and commitment to the AKI section in general. Twelve members were available and accepted the invitation. All participants were experts in the field of Critical Care Nephrology and research active. A core group of 4 members organized the meeting and agreed by consensus that the focus of the meeting should be to identify research topics in the 3 key areas of Critical Care Nephrology: “AKI diagnosis and evaluation”, “Medical management of AKI” and “Renal Replacement Therapy for AKI”. Preceding the meeting, the participants were allocated to one of the three subgroups. The methodology and the structure of the meeting were agreed in advance by the core group and discussed with the participants in order to allow sufficient time for preparation.

### ART meeting

The meeting consisted of a one-day workshop which was held in October 2018, 1 day before the annual ESICM congress. Participants were asked to self-fund their attendance. Importantly, there was no involvement or support from commercial companies. The structure of the meeting followed a modified Delphi approach. Following an initial introduction and plenary session outlining the objectives of the meeting, the participants presented the outcome of their pre-meeting work and subsequently worked in their allocated groups and developed potential proposals for future research. During two open panel discussions, each group presented their preliminary and final proposals to the other two groups for feedback, approval, and consensus. All responses and feedback were non-anonymous.

### Objectives

The aims of the ART meeting were to identify important areas where evidence and consensus were lacking and clinical practice was variable and to develop projects for future clinical research. Key requirements were that the proposals focussed on clinical aspects, were relevant to the majority of AKI section members and beyond, allowed centres from different countries to participate, and that they met global gaps in knowledge. The groups were encouraged to refer to the intensive care medicine agenda on AKI, as published in 2017 [7]. Each subgroup was asked to consider a systematic review or meta-analysis, an observational cohort study, and a prospective interventional trial, if possible. It was agreed that proposals needed to be pragmatic, multicentre, collaborative, and feasible without the need for extensive funding. There was full agreement that all members of the AKI section would be invited to collaborate and that their contribution would be acknowledged in future publications.

## Results

Following detailed review of the existing literature and in-depth discussions, the working groups identified and developed the following proposals:

### A. Group 1: Diagnosis and evaluation of AKI

The reported incidence of AKI is highly variable [6]. This heterogeneity might be explained by the definitions used (RIFLE, AKIN or KDIGO criteria), differences in patient populations, case-mix, and clinical setting as well as differences in managing missing data. Furthermore, the current consensus definition of AKI includes the notion of a continuum of disease but there is clear evidence of different sub-categories of AKI, for instance, rapid reversal AKI (duration < 48 h), persistent AKI (duration between 2 and 7 days) and acute kidney disease (up to 90 days). To the best of our knowledge, there are only limited data evaluating the epidemiology and outcomes of patients with AKI of different durations (using current criteria, including AKI stages) [9].

i. Proposal for systematic review

Aim: to summarize the existing data related to the epidemiology of AKI with stratification by different patient populations and diagnostic criteria in order to increase granularity

Endpoints: Primary endpoint: overall AKI prevalence; Secondary endpoints: AKI prevalence stratified by diagnostic criteria, population type and management of missing data.

Statistical Analysis: meta-regression and multivariable meta-regression

Criteria for study selection: (a) Publication date after 2012; (b) Publication language: English; (c) Study design: multicentre or > 100 patients recruited from the same center; and (d) Published as a full paper (no abstract)

Search Strategy: Search in Pubmed and Embase using the following MESH criteria: “Acute kidney injury” or “AKI”

ii. Proposal for Prevalence Study

Aim: to explore the epidemiology and outcome of critically ill patients according to AKI duration.

Methods: Observational retrospective multicentre prevalence study including 10 to 20 centres.

Eligibility criteria: Inclusion criteria: all patients admitted to a participating ICU during the study period (1 month); Exclusion criteria: end-stage renal disease treated with chronic dialysis or renal transplant

Definitions: AKI will be defined by the KDIGO criteria (> 26.5 μmol/l or 1.5× baseline rise in serum creatinine and/or urine output < 0.5 ml/kg/hr for 6 h) [8]; AKI will be stratified according to the duration of the alteration [persistence of AKI criteria or need for renal replacement therapy (RRT)].

Endpoints: Primary endpoint: prevalence of AKI according to duration category; Secondary endpoints: specific mortality for each AKI duration category and renal recovery at 90 days (absence of AKI criteria and need for RRT)

### B. Group 2: Medical management of AKI

To date, the management of AKI patients focuses on correction of the underlying cause, avoidance of further renal insults, strategies to prevent progression to a more severe stage of AKI and if possible, facilitation of renal recovery. A key component of this approach is haemodynamic optimization [8]. However, there is uncertainty about the exact haemodynamic targets.

The current Kidney Disease Improving Global Outcomes (KDIGO) guideline and the ESICM recommendations for “Prevention of acute kidney injury and protection of renal function in the intensive care unit” advise to consider haemodynamic monitoring early and to aim for a mean arterial pressure (MAP) greater than 65 mmHg to prevent AKI [8, 10]. Concern has been raised that this target may be too low for individual patient groups. In patients with septic shock, for instance, a retrospective study concluded that a higher target MAP was associated with better kidney function [11]. Similarly, a sub-group analysis of a randomized controlled trial (RCT) comparing different MAP targets suggested that the rate of RRT in patients with pre-existing hypertension was significantly lower if randomized to the higher blood pressure group [12]. Among patients with vasopressor-dependent shock post-cardiac surgery, those with progression of AKI had a greater difference (deficit) in hemodynamic pressure-related parameters between baseline and within-ICU stay compared to those without AKI progression [13]. Finally, there are also data suggesting that a higher MAP in critically ill patients with early AKI is associated with a reduced risk of progression to more severe AKI [14]. It is our hypothesis that a higher target MAP in critically ill patients with pre-existing hypertension is associated with better renal outcomes.

i. Proposal for systematic review and meta-analysis
  Objective: To explore whether a higher target MAP in patients at risk of AKI admitted to ICU with known hypertension is associated with improved outcomes
  Search criteria: a) RCTs only, including indirect data from RCTs; b) published after 2004; c) published in all languages
  Search engines: bibliographic databases (MEDLINE, Embase, Cochrane Library, CINAHL and Web of Science) from January 2004 to January 2018
  Patient population: adult patients (≥18 years) in Critical Care or ICU
  Definitions: a) AKI as defined by RIFLE, AKIN or KDIGO criteria; b) Hypertension as defined by criteria used in the individual studies.
ii. Proposal for randomized controlled trial
  Objective: To investigate whether a higher MAP in critically ill patients with known hypertension is renoprotective.
  Patient population: subgroup of critically ill adult patients (i.e. septic shock, post cardiac surgery or trauma; to be determined at later stage)

Eligibility criteria: Inclusion criteria: (a) admitted to a critical care unit; (b) known hypertension (i.e. hypertension treated with at least 1 antihypertensive), and c) expected to stay in ICU for at least 48 h; Exclusion criteria: a) in ICU for > 36 h; b) MAP > 70 mmHg at screening and enrolment without vasopressor support; (c) chronic kidney disease stage 4 or 5; (d) chronically dialysis dependent ESRD; (e) treatment with RRT at time of enrolment; (f) need for ECMO at time of enrolment; (g) any contraindication to higher or lower MAP target

Group allocation and interventions: Standard care: target MAP 65–75 mmHg for at least 48 h after randomization. Intervention group: target MAP 80–90 mmHg for at least 48 h after randomization.

Interventions to achieve MAP target: Traditional strategies, including fluids, vasopressors and/or inotropes as per judgement of clinical team. However, starches should not be used for fluid therapy.

Concomitant treatments: All other aspects of care will be according to local practice and discretion of the treating clinical team. The decision to initiate RRT will be made by the clinical team based on traditional clinical parameters.

Outcomes: Depending on whether patients already have AKI or no AKI at time of randomization:

i. In patients without AKI at randomization:
  Primary outcome: prevention of AKI in 7 days; Secondary outcomes: (a) mortality; (b) creatinine at 28 days; (c) Major adverse kidney event (MAKE) at 28 days; (d) hospital length of stay; (e) if AKI develops: duration and severity of AKI, including treatment with RRT; f) adverse events; (g) max dose and duration of catecholamine treatment
ii. In patients with AKI at enrolment and no immediate need for RRT at randomization
  Primary outcome: MAKE at 28 days; Secondary outcomes: (a) mortality; (b) creatinine at 28 days; (c) hospital length of stay; (d) duration and severity of AKI; (e) treatment with RRT; (f) adverse events; (g) max dose and duration of catecholamine treatment

Potential challenges: The main limitation is the fact that the intervention strategies to raise MAP and all other concomitant therapies are not standardized. As such, the outcomes may be influenced by factors other than target MAP, for instance choice and volume of fluids, use of nephrotoxic drugs and decision to initiate RRT.

### C. Group 3: Renal replacement therapy for AKI

The optimal modality of continuous renal replacement therapy (CRRT) for AKI is unknown and clinical practice is highly variable [15, 16]. To date, precise and exhaustive information about how CRRT is administered worldwide in 2020 is lacking. There is no clear evidence that specific modalities of CRRT are superior to others, and detailed analyses of the effects of CRRT modality on solute clearance are limited [17–20]. It is assumed that continuous venovenous haemofiltration (CVVH) leads to better clearance of larger molecules compared to continuous venovenous haemodialysis (CVVHD) due to solutes being dragged by the solvent. However, this has not been confirmed [21, 22]. In addition, clearance of larger molecules in CVVH may be impacted with time by clogging related to hemoconcentration inside the filter (depending on the filtration fraction). Lastly, there are no recent data about the clinical effects of convection versus diffusion in CRRT.

i. Proposal for systematic review and meta-analysis

Aim: To systematically search and compile the available data focusing on the comparison of CVVH versus CVVHD

Endpoints: Primary endpoint: Solute clearance of various molecules, cytokines and antibiotics*;* Secondary endpoints: clinical endpoints

Study Selection: (a) studies comparing CRRT with diffusion (CVVHD) versus CRRT with convection (CVVH); (b) published as full papers; (c) published in English

ii. Proposal for observational study

Aim: to capture data related to the current clinical practice of CRRT

Design: International survey sent to institutions worldwide (using ESICM research network). Methodology: Questions will be primarily focused on CRRT modality to explore the use of CVVH, CVVHD and CVVHDF. Additional questions will intend to describe how these 3 modalities are administered in daily clinical practice (dose, anticoagulation, timing, type of hemofilter, average filter duration, compulsory change of set every 24 h (yes/no), number of treatment days per year or number of treated patients/year).

iii. Proposal for interventional study

Hypothesis: Solute clearance is similar between CVVH and CVVHD, including for middle molecular weight solutes

Aim: To compare clearances of different solutes with various molecular weights between CVVH and CVVHD over 72 h

Inclusion criteria: Critically ill patients undergoing CRRT

Design: Patients will be randomized in two groups, CVVH or CVVHD. CRRT dose and anticoagulation (citrate) will be the same in both groups, based on current KDIGO recommendations [8]. Membranes will be polysulfone-based (e.g. HF1400, AV1000S) with a standard cut-off of 30,000 Da. Solute clearances will be calculated at the following time-points: 1 h, 6 h, 12 h, 24 h, 48 h, 72 h. Cross-over will not be allowed.

Outcomes: Primary outcome: clearance of one large molecular solute (e.g. beta 2 microglobulin); Secondary outcomes: a) time weighted average solute clearances including urea, creatinine, Interleukin-6, tumor necrosis factor (TNF), kappa Free Light Chain (25,000 Da), lambda Free Light Chain (50,000 Da), albumin; b) Metabolic endpoints: acid-base status and electrolyte disturbances; c) Hemodynamic impact endpoint: vasopressor treatment; d) filter life; e) survival at 30 day and hospital discharge; f) CRRT free days; g) Renal recovery: dialysis dependence at hospital discharge; h) filter survival time

Number of patients per group: 60 patients

Additional remarks: the study will only be conducted in centres where both modalities (CVVH and CVVHD) can be delivered in order to minimize potential confounders.

## Discussion

The first ART meeting identified and developed key proposals in the fields of epidemiology, medical management and RRT. In Critical Care Nephrology, there is a clear need for larger epidemiological research but also small, well defined studies addressing specific niche aspects, like clearance during CRRT.

The current definition of AKI is very pragmatic but does not account for the heterogeneity of AKI as a syndrome [8]. Instead of describing a specific pathophysiological entity, AKI represents an “umbrella” term for a vast number of conditions characterized by a rise in serum creatinine or decrease in urine output. In addition, important aspects related to AKI, including recurrence and clinical context are not incorporated in the current criteria. Finally, the significance of duration of AKI remains uncertain. This includes short periods of AKI or rapid reversal of AKI, or AKI lasting for 7 days or longer, so called “Acute Kidney Disease” (AKD) [8, 23].

The proposals of the working group on diagnosis and definition of AKI will focus on the epidemiology of AKI in different subgroups of critically ill patients and its etiology and duration. As such, they will explore the epidemiology and outcomes according to the current KDIGO definition and investigate the impact of different patterns and durations of AKI [23]. Especially, the two types “rapid reversal of AKI“ (i.e. duration of 48 h or less) and “AKD” (duration up to 90 days) have not been well explored so far. Finally, the nature of the ESICM AKI network may also allow an investigation of regional differences in the epidemiology of AKI.

To date, the management of AKI remains supportive with focus on correction of volume depletion and hypotension, avoidance of further nephrotoxic insults and treatment of the underlying acute illness. Although this makes sense at first glance, the details for management at the bedside are scarce or lacking [24]. For instance, there is no consensus on the optimal method of assessing volume status, rate of fluid administration, haemodynamic targets to prevent or reverse AKI and the optimal strategy to correct hypotension. Accordingly, the ART panel identified blood pressure as the target of a pragmatic and feasible future intervention study.

Finally, clinical trials in the field of RRT have established that a higher dose of RRT does not translate into better outcomes and that regional anticoagulation with citrate is associated with longer filter life in CRRT. However, other important aspects are still uncertain, for instance the optimal modality, timing and discontinuation of RRT [16, 24]. Despite several decades of applying convective and diffusive RRT modalities, the evidence on its use is very limited. At present, the application of diffusive and convective therapies is mainly driven by pragmatic arguments, and not by evidence. Since clearance is an essential element of RRT, the RRT working group selected this topic for further studies: a literature review, an international survey and a randomized controlled trial. If successful, we believe the results will inform future decision making in Critical Care Nephrology.

It is fully acknowledged that there are many more areas where clinical practice is variable and more evidence is urgently required. The suggested proposals by the ART panel members represent areas of high priority and allow participation and collaboration among all interested ESICM AKI section members and beyond.

In conclusion, the ART panel identified pragmatic and feasible studies that are open to all ESICM AKI section members to participate. These studies cover the entire spectrum of clinical AKI in 3 domains: diagnosis and epidemiology, medical therapy and RRT. Although achieving consensus among experts is already a major achievement, we acknowledge that more work is necessary to develop the relevant protocols further, secure the necessary funding, and recruit collaborators.

The ART meeting was the first of hopefully a series of meetings which aim to develop and facilitate collaborative research to advance the field of Critical Care Nephrology and improve the outcome of patients with AKI.

## Data Availability

Not applicable.
